# Supplementary material for: Lipopolysaccharide binding protein resists hepatic oxidative stress by regulating lipid droplet homeostasis
Source: Nat Commun. 2024 Apr 13;15:3213. doi: 10.1038/s41467-024-47553-5 (PMC11016120; doi:10.1038/s41467-024-47553-5)
Supplement: Supplementary file 1 — Supplementary Information [file 41467_2024_47553_MOESM1_ESM.pdf]

Supplementary Information

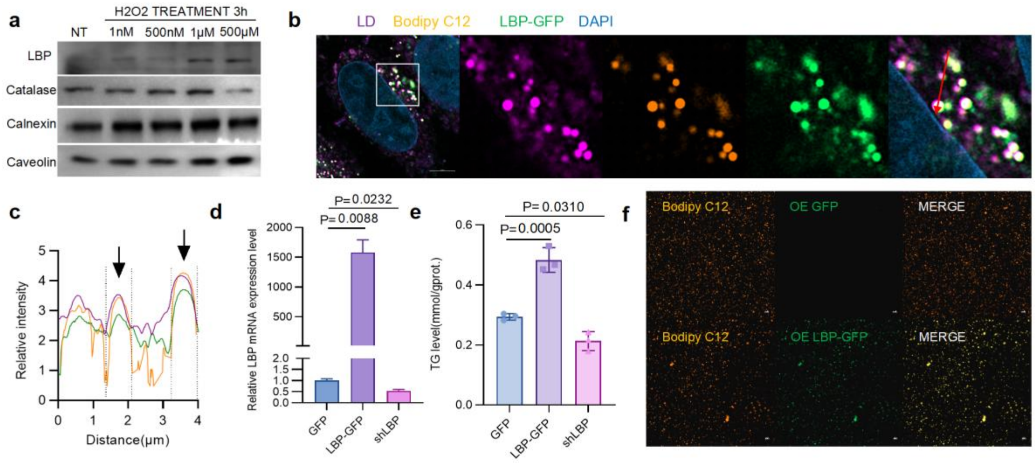

Supplementary Fig.1 LBP localizes to LDs and promotes LD enlargement and TG accumulation in HepG2 cells.

(a) Dose-dependent upregulation of LBP in HepG2 cells after treatment with H_2O_2 for 3 hours.

(b) Representative photographs displaying colocalization of LBP-GFP with Bodipy C12 and LDs in HepG2 cells, scale bar=5μm. Five independent experiments with similar results.

(c) Quantitative analysis of the relative intensity of colocalization in panel (b).

(d) LBP mRNA expression level in HepG2 cells with LBP overexpression and knockdown. Shown are means±s.d., unpaired t-test (n=2-3, biologically independent).

(e) TG levels in HepG2 cells with LBP overexpression and knockdown after treatment with Bodipy C12 for 24 hours. Shown are means±s.d., unpaired t-test (n=3, biologically independent).

(f) Representative confocal images of LDs extracted from HepG2 cells overexpressing GFP/LBP-GFP and treated with Bodipy C12 for 24 hours, scale bar=5μm. Three independent experiments with similar results.

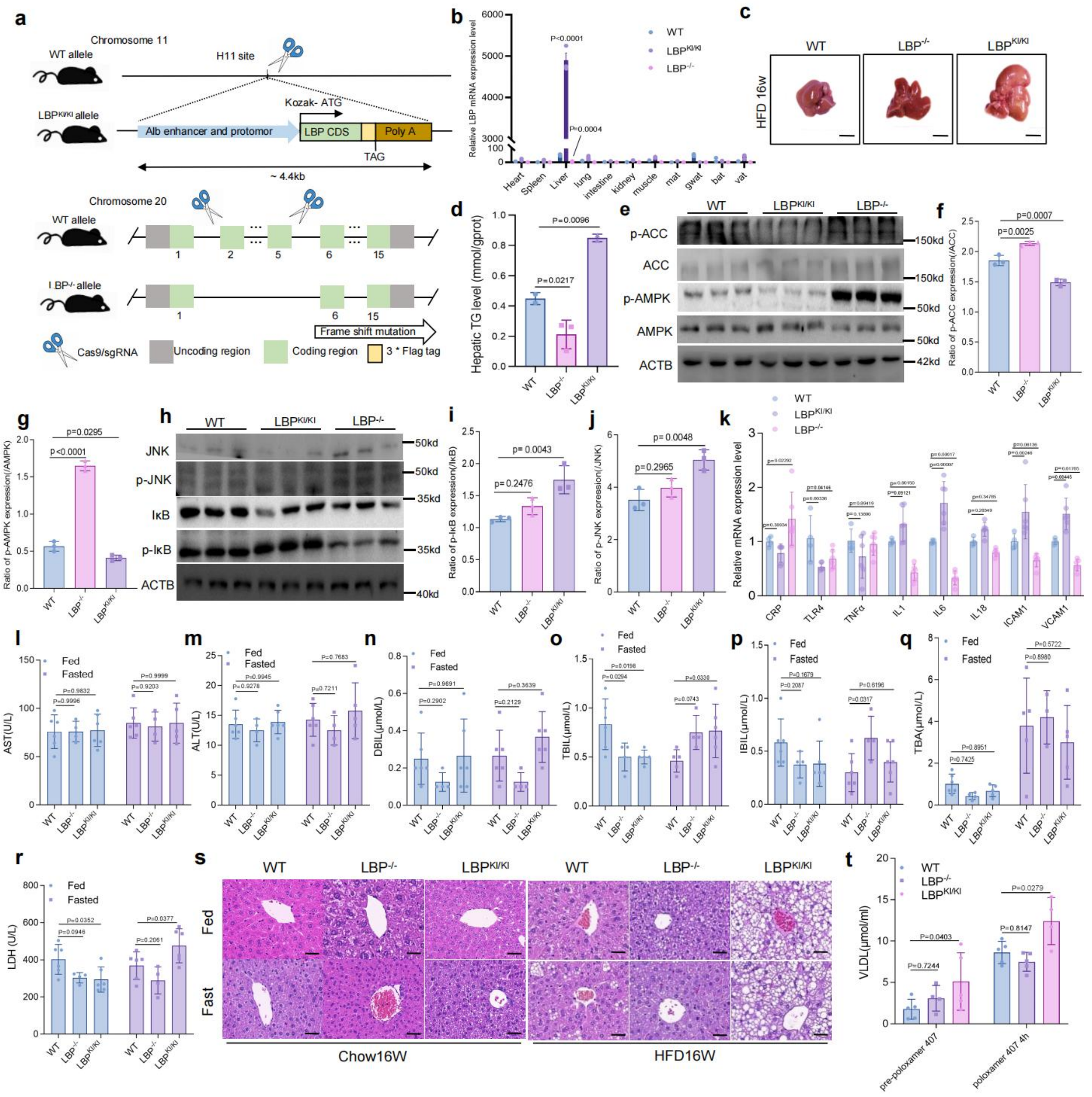

Supplementary Fig.2 Pathological and physiological indicators of liver function in WT, LBP$^{-/-}$ and LBP$^{KI/KI}$ mice.

(a) Schematic illustration of LBP$^{-/-}$ and LBP$^{KI/KI}$ mice construction. Illustrated by Ying Mei. Permission has been granted.

(b) LBP mRNA expression levels in peripheral tissues of WT, LBP$^{-/-}$ and LBP$^{KI/KI}$ mice. Shown are means±s.d., unpaired t-test (n=3, biologically independent).

(c) Representative photographs of WT, LBP$^{-/-}$ and LBP$^{KI/KI}$ livers after HFD for 16 weeks (scale bar=1cm).

(d) Quantification of TG levels in liver tissue of each group in (c). Shown are means±s.d., one-way ANOVA (n=2-3, biologically independent).

(e) The WB assay showed the expression levels of p-AMPK and p-ACC in the three genotypes of mice in (c).

(f-g) Quantification of p-AMPK and p-ACC levels in (e). Shown are means±s.d., one-way ANOVA (n=3, biologically independent).

(h) The WB assay showed the expression levels of p-JNK and p-IκB in the three genotypes of mice in (c).

(i-j) Quantification of p-JNK and p-IκB levels in (h). Shown are means±s.d., one-way ANOVA (n=3, biologically independent).
(k) The bar graph displays the results of the qPCR analysis, revealing the expression levels of inflammation-related genes. Shown are means±s.d., 2way ANOVA (n=4-6, biologically independent).
(l-r) Levels of serum liver function indexes: AST, ALT, DBIL, TBIL, IBIL, TBA and LDH in WT, $LBP^{-/-}$ and $LBP^{KI/KI}$ mice after feeding and fasting. Shown are means±s.d., 2way ANOVA (n=4-6, biologically independent).
(s) Liver tissue HE staining (scale bar=20μm). Experiments was conducted with three biological replicates and produced similar results.
(t) Serum VLDL levels of WT, $LBP^{-/-}$ and $LBP^{KI/KI}$ mice before and after poloxamer 407 injection. Shown are means±s.d., 2way ANOVA (n=4-5, biologically independent).

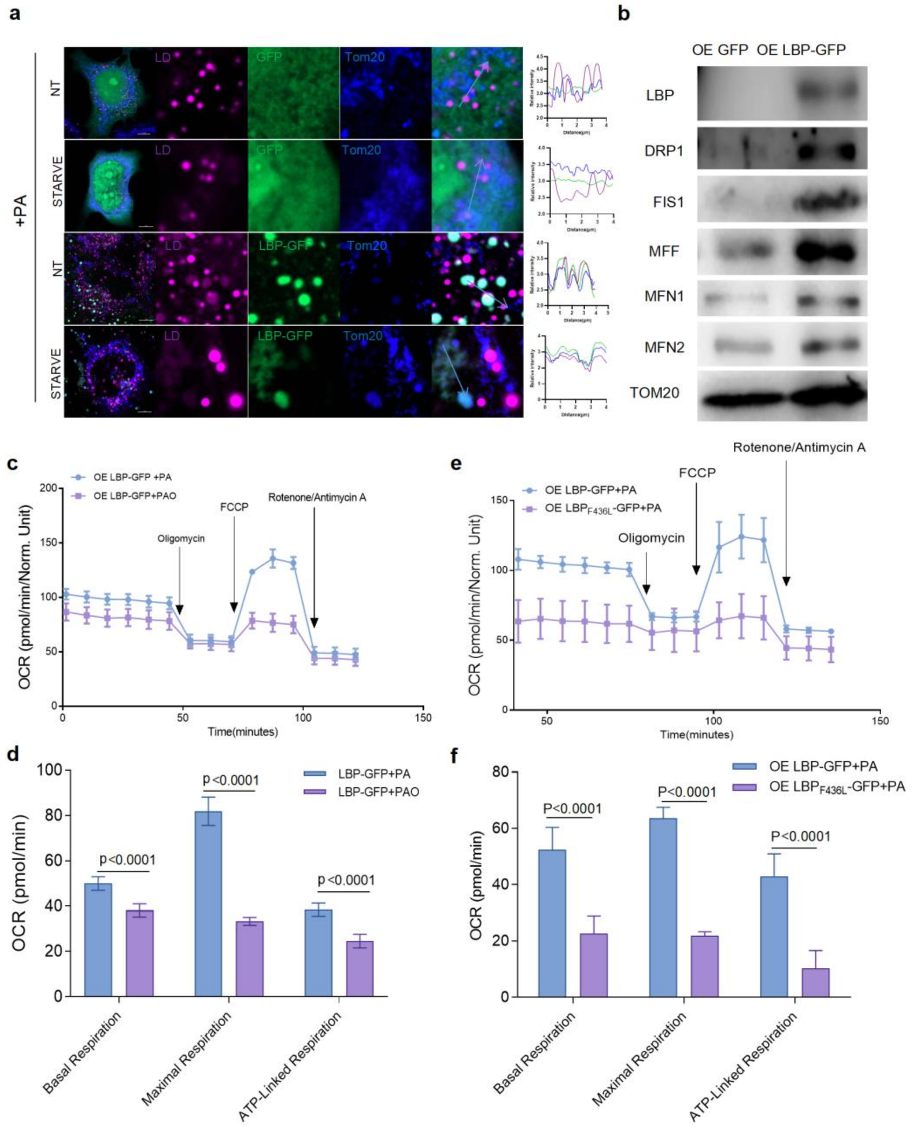

Supplementary Fig.3 LBP promotes PA metabolism in mitochondria.

(a) Staining images of Tom20 and Bodipy 665/675 in HepG2 cells overexpressing LBP-GFP for 24 hours and treated with 100 μM PA for 24 hours, followed by nutritional-starvation for 3 hours, scale bar=5μm. Experiments repeated two times independently with similar results.

(b) Expression levels of mitochondrial fusion and division-related proteins in cells from panel (a).

(c) Kinetic line graph of seahorse analysis demonstrating that LBP promotes the degradation of saturated fatty acids, rather than unsaturated fatty acids. Shown are means±s.d. (n=3, biologically independent).

(d) Analysis of basal, maximal OCR, and ATP-linked respiration data in (c). Shown are means±s.d., 2way ANOVA (n=3, biologically independent).

(e) Kinetic line graph of seahorse analysis demonstrating that the F436L mutation disables the function of LBP in protecting PUFA-TG. Shown are means±s.d. (n=4, biologically independent)..

(f) Analysis of basal, maximal OCR, and ATP-linked respiration data in (e). Shown are means±s.d., 2way ANOVA (n=4, biologically independent).

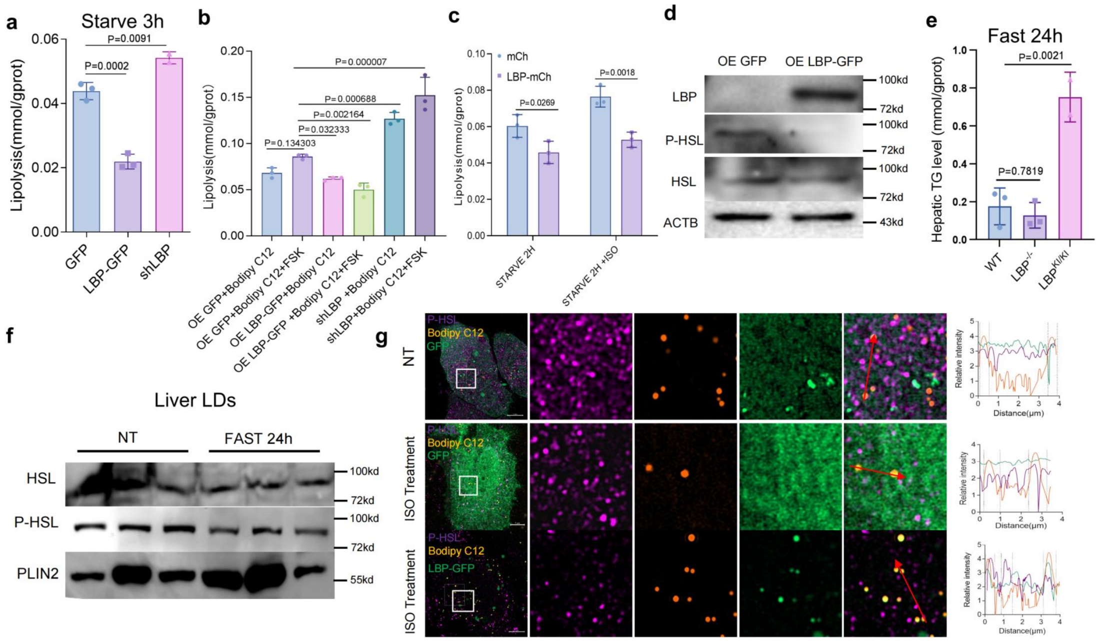

Supplementary Fig.4 LBP suppresses lipolysis by impacting P-HSL function.

(a) FFA assay demonstrate that LBP overexpression inhibits the lipolysis induced by starvation for 3h, while shLBP has the opposite effect. Shown are means±s.d., one-way ANOVA (n=2-3, biologically independent).

(b) LBP overexpression inhibits FSK-stimulated lipolysis, while shLBP has the opposite effect. Shown are means±s.d., one-way ANOVA (n=3, biologically independent).

(c) A bar graph indicating that overexpression of LBP-mCherry inhibits ISO-stimulated lipolysis. Shown are means±s.d., 2way ANOVA (n=3, biologically independent).

(d) Western blot data showing that overexpression of LBP decreases P-HSL levels after ISO-stimulated lipolysis in HepG2 cells.

(e) Liver TG levels were measured in WT, $LBP^{-/-}$ and $LBP^{KI/KI}$ mice after 24h fasting. Shown are means±s.d., one-way ANOVA (n=2-3, biologically independent).

(f) LD HSL and phosphorylated HSL levels in NT/fasting 24h mice liver.

(g) Confocal microscopy images of GFP/LBP-GFP, Bodipy C12, and P-HSL co-staining after 100nM ISO treatment for 30 minutes, scale bar=5μm. Experiments repeated two times independently with similar results.

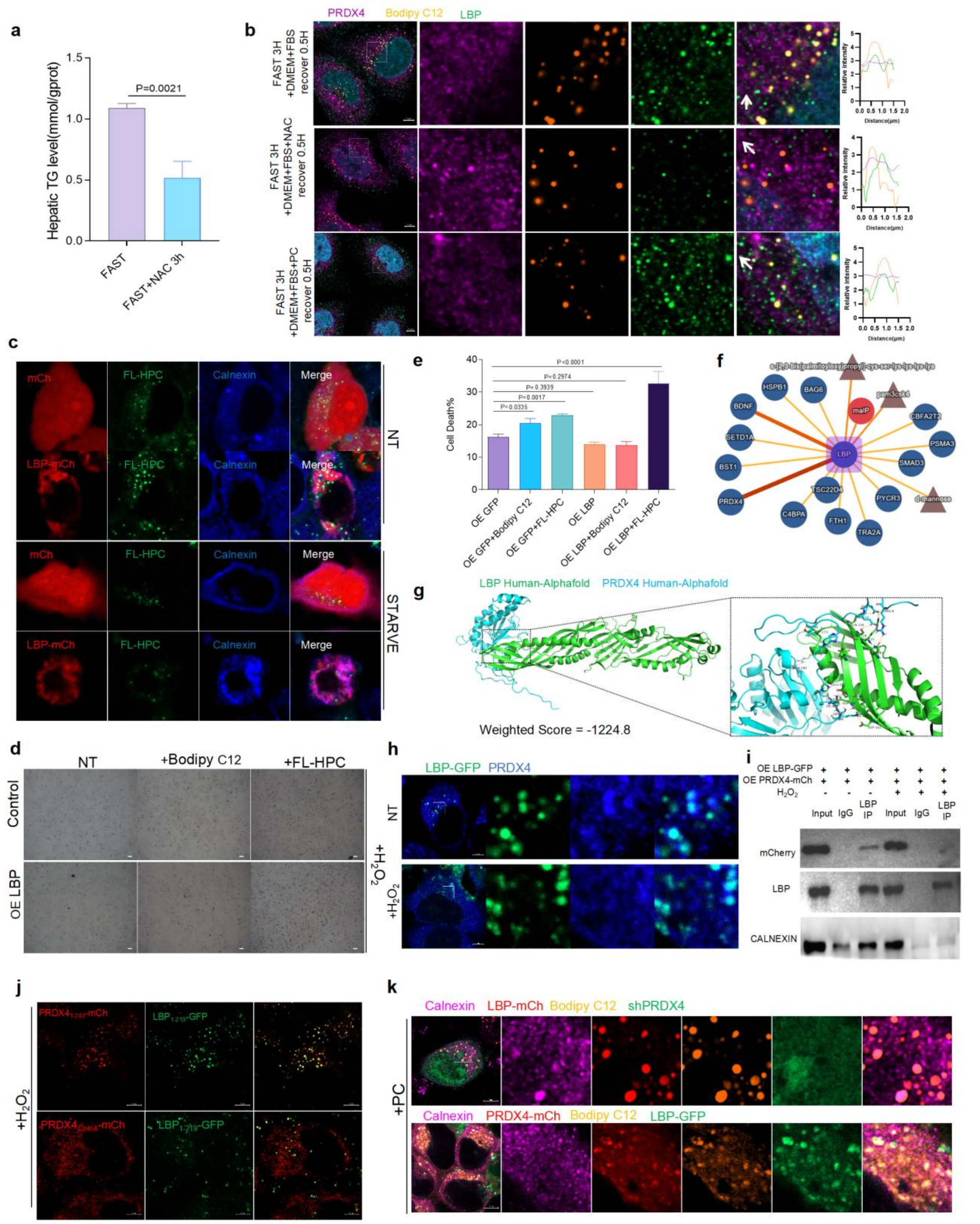

Supplementary Fig. 5 The interplay between LBP, PC and PRDX4 dictates its association with LDs.

(a) Analysis of hepatic TG levels in 8-week-old WT mice fasted for 24h (n=3), and treated with NAC for 3h. Shown are means±s.d., unpaired t-test (n=3, biologically independent).

(b) Confocal microscopy images of LBP and PRDX4 co-staining in HepG2 cells treated with Bodipy C12 for 24 hours, then starved for 3 hours. Cells were subsequently treated with complete medium, 20μM NAC or 2μM PC for rescue, scale bar=5μm. Experiments repeated three times independently with similar

results.
(c) Representative images of confocal co-localization of LBP and Calnexin in cells treated with FL-HPC for 24 hours, scale bar=5μm. Experiments repeated three times independently with similar results.
(d) Trypan blue staining images of HepG2 cells overexpressing LBP for 24 hours, followed by treatment with Bodipy C12 or FL-HPC for 24 hours. The cells were then treated with 1mM H_2O_2 for 3 hours, scale bar=100μm. Experiments repeated three times independently with similar results.
(e) Statistical data of cell death in (d). Shown are means±s.d., one-way ANOVA (n=3, biologically independent).
(f) Possible protein-protein interactions with LBP according to the EBI database (www.ebi.ac.uk), with thicker red lines indicating stronger reliability.
(g) Molecular docking of LBP human-alphfold and PRDX4 human-alphfold by Cluspro web server (https://cluspro.bu.edu).
(h) Representative images of LBP-GFP co-stained with PRDX4, before and after the addition of 500μM H_2O_2, scale bar=5μm. Experiments repeated three times independently with similar results.
(i) In vitro co-immunoprecipitation experiments of LBP-GFP and PRDX4-mCherry before and after treatment with 500μM H_2O_2 for 2 h.
(j) Typical image of $PRDX4_{C245A}$-mCh and $PRDX4_{1-243}$-mCh co-stained with LBP_{1-219}-GFP, scale bar=5μm. Experiments repeated three times independently with similar results.
(k) Co-localization of exogenous LBP, calnexin and Bodipy C12 after shPRDX4 treatment (upper panel) and overexpression of PRDX4-mCherry (lower panel), scale bar=5μm. Experiments repeated three times independently with similar results.

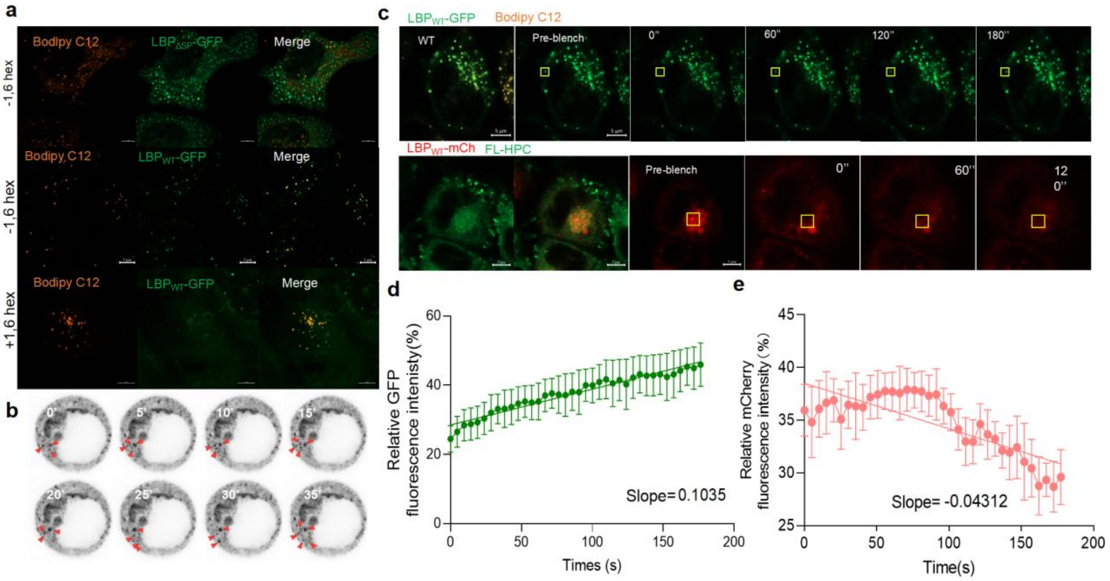

Supplementary Fig. 6 Hydrophobic forces regulate the interaction between LBP and lipid droplets.

(a) Representative photographs of Bodipy C12 and $LBP_{\Delta SP}$-GFP/LBP_{WT}-GFP fluorescence in HepG2 cells before and after adding 1,6 hexanediol for 15 seconds, scale bar=5μm. Experiments repeated five times independently with similar results.

(b) Motility of LBP-GFP droplets. Red arrows indicate fusing or dividing LBP-GFP droplets.

(c) Representative images of FRAP analysis measuring the mobility of LBP_{WT}-GFP/LBP_{WT}-mCherry. Bodipy C12/FL-HPC was introduced concomitantly with transfection and observed 20h later. The yellow box highlights the punctum undergoing targeted bleaching, scale bar=5μm.

(d-e) The quantitative line graphs of (c).

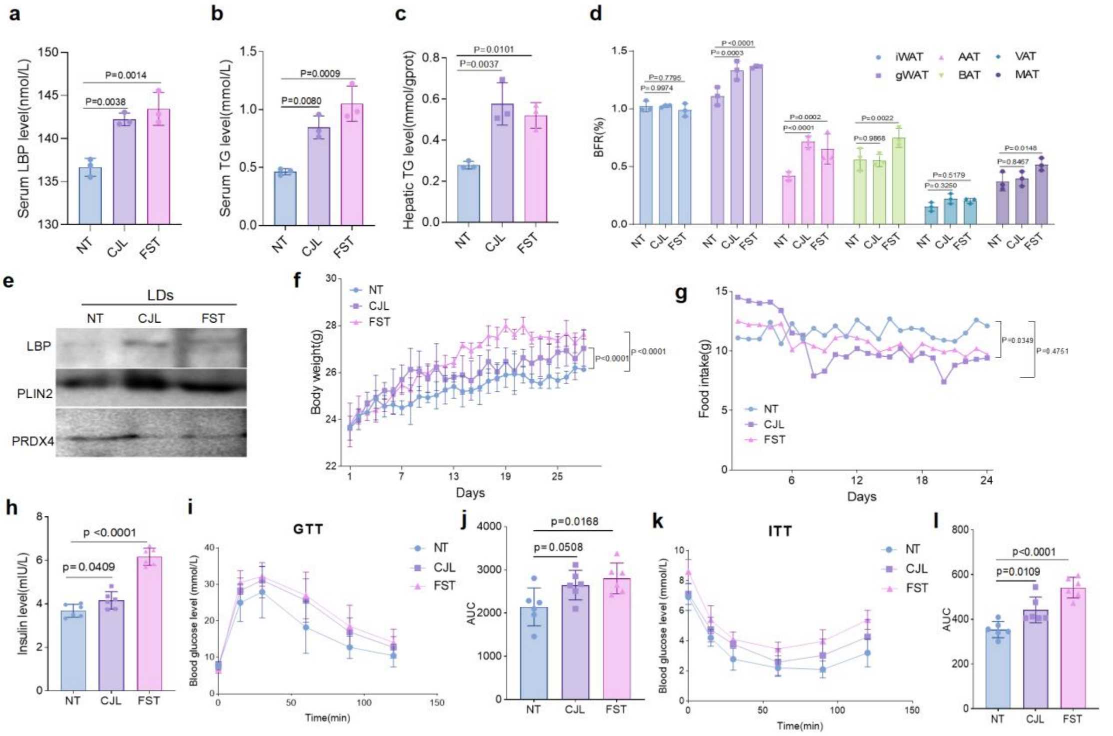

Supplementary Fig. 7 Chronic stress causes LBP-mediated obesity.

(a) LBP levels in chow-diet WT mice peripheral blood after NT, CJL and FST for 1 month. Shown are means±s.d., one-way ANOVA (n=3, biologically independent).

(b) TG levels in chow-diet WT mice peripheral blood after NT, CJL and FST for 1 month. Shown are means±s.d., one-way ANOVA (n=3, biologically independent).

(c) Liver TG levels in chow-diet WT mice after NT, CJL and FST for 1 month. Shown are means±s.d., one-way ANOVA (n=3, biologically independent).

(d) Body fat ratios in different positions of chow-diet WT mice after NT, CJL and FST for 1 month. Shown are means±s.d., 2way ANOVA (n=3, biologically independent). iWAT: Inguinal white adipose tissue, AAT: Armpit white adipose tisssue, VAT: Visceral adipose tissue, gWAT: gonadal adipose tissue, BAT: Brown adipose tissue, MAT: Mesenteric adipose tissue.

(e) LBP and PRDX4 levels in chow-diet mice liver LDs after NT, CJL and FST for 1 month.

(f) One month of CJL and FST increased body weight in 8-week-old chow-diet mice compared to NT. Shown are means±s.d., 2way ANOVA (n=3, biologically independent).

(g) One month of CJL and FST decreased the food intake in 8-week-old chow-diet mice compared to NT. Shown are means, one-way ANOVA (n=3, biologically independent).

(h) The fasting insulin levels of the NT, FST, and CJL mice. Shown are means±s.d., unpaired t-test (n=6, biologically independent).

(i) The serum glucose levels of NT, FST and CJL mice fed a high-fat diet for 4 weeks during glucose tolerance test. Shown are means±s.d. (n=6, biologically independent).
(j) Area under curve (AUC) analysis in (i). Shown are means±s.d., unpaired t-test (n=6, biologically independent).
(k) The serum glucose levels of NT, FST and CJL mice fed a high-fat diet for 4 weeks during a insulin tolerance test. Shown are means±s.d. (n=6, biologically independent).
(l) Area under curve (AUC) analysis in (k). Shown are means±s.d., unpaired t-test (n=6, biologically independent).
