# Supplementary material for: Lipopolysaccharide binding protein resists hepatic oxidative stress by regulating lipid droplet homeostasis
Source: Nat Commun. 2024 Apr 13;15:3213. doi: 10.1038/s41467-024-47553-5 (PMC11016120; doi:10.1038/s41467-024-47553-5)
Supplement: Supplementary file 3 — Description of Additional Supplementary Files [file 41467_2024_47553_MOESM3_ESM.pdf]

Description of Additional Supplementary Files

File name: Supplementary Data 1
Description: Table Of differential protein of hepatic Lipid droplets and hepatic differential mRNA of 8-week-old mice before and after 24h fasting.

File name: Supplementary Data 2
Description: Table Of hepatic lipidomics data of WT and $LBP^{KI/KI}$ mice fed with HFD for 16 Weeks.

File name: Supplementary Data 3
Description: Table Of joint analysis of liver transcriptome and proteome data from WT and $LBP^{KI/KI}$ mice after 16 weeks of HFD.

File name: Supplementary Data 4
Description: Table Of hepatic lipidomics data of 24h fasting WT mice before and after NAC treatment.

File name: Supplementary Movie 1
Description: After 24 hours of transfection with LBP-GFP followed by treated with 1μM Bodipy C12 for 16 hours, live-cell fluorescence imaging was performed on HepG2 cells . Green dots represent LBP-GFP, while orange dots indicate lipid droplets containing Bodipy C12.
