# Supplementary material for: Lipopolysaccharide binding protein resists hepatic oxidative stress by regulating lipid droplet homeostasis
Source: Nat Commun. 2024 Apr 13;15:3213. doi: 10.1038/s41467-024-47553-5 (PMC11016120; doi:10.1038/s41467-024-47553-5)
Supplement: Supplementary file 9 — Reporting Summary [file 41467_2024_47553_MOESM9_ESM.pdf]

nature portfolio

Corresponding author(s): Haoshu Fang, Mao Zheng, Shandong Ye, Anding Liu

Last updated by author(s): Mar 10, 2024

Reporting Summary

Nature Portfolio wishes to improve the reproducibility of the work that we publish. This form provides structure for consistency and transparency in reporting. For further information on Nature Portfolio policies, see our Editorial Policies and the Editorial Policy Checklist.

Statistics

For all statistical analyses, confirm that the following items are present in the figure legend, table legend, main text, or Methods section.

n/a	Confirmed	
☐	☒	The exact sample size (*n*) for each experimental group/condition, given as a discrete number and unit of measurement
☐	☒	A statement on whether measurements were taken from distinct samples or whether the same sample was measured repeatedly
☐	☒	The statistical test(s) used AND whether they are one- or two-sided<br>*Only common tests should be described solely by name; describe more complex techniques in the Methods section.*
☐	☒	A description of all covariates tested
☒	☐	A description of any assumptions or corrections, such as tests of normality and adjustment for multiple comparisons
☐	☒	A full description of the statistical parameters including central tendency (e.g. means) or other basic estimates (e.g. regression coefficient) AND variation (e.g. standard deviation) or associated estimates of uncertainty (e.g. confidence intervals)
☐	☒	For null hypothesis testing, the test statistic (e.g. *F*, *t*, *r*) with confidence intervals, effect sizes, degrees of freedom and *P* value noted<br>*Give P values as exact values whenever suitable.*
☒	☐	For Bayesian analysis, information on the choice of priors and Markov chain Monte Carlo settings
☒	☐	For hierarchical and complex designs, identification of the appropriate level for tests and full reporting of outcomes
☐	☒	Estimates of effect sizes (e.g. Cohen's *d*, Pearson's *r*), indicating how they were calculated

Our web collection on statistics for biologists contains articles on many of the points above.

Software and code

Policy information about availability of computer code

Data collection	ZEN 2.6 (blue edition); SHST Capture(V.2.0.1.107); OD-2000C (V3.7.29); MO.Control (V.1.6.1); TEM Imaging & Analysis software (V.4.7 SP1).
Data analysis	Graphpad Prism 9.5; Image J; Snapgene (V.4.2.4); Bowtie2 (V.2.2.8); HISAT(V.2.2.4); StringTie (V.1.3.1); DESeq2 (V.1.24.0); Proteome Discoverer search engine (v2.4.1.15); LipidSearch software (V.4.2); MO Affinity Analysis (V.2.3) I-TASSER (V.5.2); Open Babel (V.2.3.1); AutoDock Vina (1.2.0); PyMOL(V2.5); XFE software (V2.6.1.56)

For manuscripts utilizing custom algorithms or software that are central to the research but not yet described in published literature, software must be made available to editors and reviewers. We strongly encourage code deposition in a community repository (e.g. GitHub). See the Nature Portfolio guidelines for submitting code & software for further information.

Data

Policy information about availability of data

All manuscripts must include a data availability statement. This statement should provide the following information, where applicable:

- Accession codes, unique identifiers, or web links for publicly available datasets
- A description of any restrictions on data availability
- For clinical datasets or third party data, please ensure that the statement adheres to our policy

The RNAseq data have been made available at the NCBI SRA repository under accession number PRJNA939362 (http://www.ncbi.nlm.nih.gov/sra). We used GRCm39 as the refseq (https://ftp.ensembl.org/pub/release-111/fasta/mus_musculus/dna/). The mass spectrometry proteomics data have been deposited to

PRIDE database under accession number PXD040940 (http://www.ebi.ac.uk/pride). We conducted protein database searching using the Uniport mouse FASTA database Mus_musculus_10090_SP_20220107.fasta(https://www.uniprot.org/taxonomy/10090). Metabolicdata were uploaded to Metabolomics Workbench under accession number ST002522 (https://www.metabolomicsworkbench.org). All relevant data are available from Figshare (10.6084/m9.figshare.22187665).

Research involving human participants, their data, or biological material

Policy information about studies with human participants or human data. See also policy information about sex, gender (identity/presentation), and sexual orientation and race, ethnicity and racism.

Reporting on sex and gender	N/A
Reporting on race, ethnicity, or other socially relevant groupings	N/A
Population characteristics	N/A
Recruitment	N/A
Ethics oversight	N/A

Note that full information on the approval of the study protocol must also be provided in the manuscript.

Field-specific reporting

Please select the one below that is the best fit for your research. If you are not sure, read the appropriate sections before making your selection.

☒ Life sciences ☐ Behavioural & social sciences ☐ Ecological, evolutionary & environmental sciences

For a reference copy of the document with all sections, see nature.com/documents/nr-reporting-summary-flat.pdf

Life sciences study design

All studies must disclose on these points even when the disclosure is negative.

Sample size	The experimental design, including the calculation strategy, was formulated in accordance with the FELASA B class material (2017) as outlined by Friedrich-Schiller-Universität Jena. The chosen sample size was determined to ensure sufficient statistical significance while adhering to principles of animal welfare, with a particular emphasis on the 3R approach (replacement, reduction, and refinement). To evaluate the cumulative impact, multiple time points were analyzed, including 9 and 16 weeks of high-fat diet exposure. These evaluations consistently revealed that LBP induces hepatic steatosis by facilitating the accumulation of unsaturated triglycerides. Our findings were further validated through cell-based experiments that consistently produced affirming results. The cumulative use of these methodologies enhances the confidence in the dependability and authenticity of our data.
Data exclusions	No data were excluded from the analyses. All collected data were included in the study.
Replication	The number of repetitions for the experiments has been described in the figure legends. All attempts at replication were successful.
Randomization	In this study, samples were allocated into experimental groups using a randomization procedure. Furthermore, covariates such as age, gender and enviromental factors were controlled during the allocation process to account for any factors that may have influenced the outcomes of interest.
Blinding	We used blinding in some experiments, such as ear tagging mice of different genotypes and feeding them a high-fat diet, which led to the conclusion that LBP can induce obesity and hepatic steatosis in mice. However, in some other experiments, blinding was not possible. For example, in the comparison of mice before and after fasting, drug interventions, or FST and CJL experiments, we needed to know the group allocation to ensure the proper execution of the experiments.

Reporting for specific materials, systems and methods

We require information from authors about some types of materials, experimental systems and methods used in many studies. Here, indicate whether each material, system or method listed is relevant to your study. If you are not sure if a list item applies to your research, read the appropriate section before selecting a response.

Materials & experimental systems

n/a	Involved in the study
☐	☒ Antibodies
☐	☒ Eukaryotic cell lines
☒	☐ Palaeontology and archaeology
☐	☒ Animals and other organisms
☒	☐ Clinical data
☒	☐ Dual use research of concern
☒	☐ Plants

Methods

n/a	Involved in the study
☒	☐ ChIP-seq
☒	☐ Flow cytometry
☒	☐ MRI-based neuroimaging

Antibodies

Antibodies used

LBP Polyclonal antibody (Cat.#23559-1-AP, Lot.#00017794), LBP Monoclonal antibody (Cat.#66181-1-Ig, Lot.#10001902), Calnexin Polyclonal antibody (Cat.#10427-2-AP, Lot.#00093709), HSL Polyclonal antibody (Cat.#17333-1-AP, Lot.#00055498), ADRP/Perilipin 2 Polyclonal antibody (Cat.#15294-1-AP, Lot.#00093566), PRDX4 Polyclonal antibody(Cat.#10703-1-AP, Lot.#00051410), TOM20 Monoclonal antibody (Cat.#66777-1-Ig, Lot.#10020635), CoraLite488-conjugated Goat Anti-Mouse IgG(H+L) (Cat.#SA00013-1, Lot.#20000422), CoraLite594-conjugated Goat Anti-Rabbit IgG(H+L) (Cat.#SA00013-4, Lot.#20000239), HRP-conjugated Affinipure Goat Anti-Rabbit IgG(H+L) (Cat.#SA00001-2, Lot.#20000798) and HRP-conjugated Affinipure Goat Anti-Mouse IgG(H+L) (Cat.#SA00001-1, Lot.#20000325) were purchased from Proteintech Inc.; Phospho-HSL (Ser660) Antibody (Cat.#AF8026, Lot.#40u3105) was from Affinity Biosciences; Anti-Catalase Mouse mAb (Cat.#PTM-5630, Lot.#ML070144), Anti-Caveolin-1 Rabbit mAb (Cat.#PTM-5056, Lot.#L030327) were from PTM Bio Inc.; β-actin Mouse Monoclonal Antibody (Cat.#E12-041, Lot.#EG20200316) and mCherry-Tag mouse monoclonal antibody (Cat.#E12-010, Lot.#EG20200620) were from EnoGene Biotech Inc.; Goat Anti Rabbit IgG (H&L)-Alexa Fluor 647(Cat.#RS3811, Lot.#B1101RA13) was from Immunoway; p-AMPKα(T182/T172) antibody (Cat.#WL05103, Lot.#R08215103), AMPKα antibody (Cat.#WL02254, Lot.#R08232254), IκBα antibody (Cat.#WL01936, Lot.#R08241936), p-IκBα (ser32/ser36) antibody (Cat.#WL02495, Lot.#R06132495), JNK antibody (Cat.#WL01295, Lot.#L03041295), p-JNK (Thr183/Tyr185) antibody (Cat.#WL01813, Lot.#L03161813) were from Wanleibio Inc.; Goat Anti-Mouse IgG H&L / AF350 antibody (Cat.#bs-0296G-AF350, Lot.#AI04125842)was from Bioss Inc.

Validation

LBP Polyclonal antibody (23559-1-AP):IF, IP, WB,ELISA; LBP Monoclonal antibody (66181-1-Ig):IP,IF, IHC, WB, ELISA; Calnexin Polyclonal antibody (10427-2-AP): FC, IF, IHC, WB, ELISA; HSL Polyclonal antibody (17333-1-AP): IF, IHC, IP, WB; ADRP/Perilipin 2 Polyclonal antibody (15294-1-AP): CoIP, IF, IHC, WB, ELISA; PRDX4 Polyclonal antibody(10703-1-AP): FC, IF, IHC, IP, WB, ELISA; TOM20 Monoclonal antibody (66777-1-Ig): FC, IF, IHC, WB, ELISA; Phospho-HSL (Ser660) Antibody (AF8026): WB, IHC, IF/ICC; Anti-Catalase Mouse mAb (PTM-5630): WB, IHC-P, IF; Anti-Caveolin-1 Rabbit mAb (PTM-5056): WB,IHC-P; β-actin Mouse Monoclonal Antibody (E12-041): WB, IF, IHC; mCherry-Tag mouse monoclonal antibody (E12-010): WB, IP; p-AMPKα(T182/T172) antibody (WL05103): WB, IHC,IF; AMPKα antibody (WL02254):WB,IHC,IF; IκBα antibody (WL01936):WB, IHC; p-IκBα (ser32/ser36) antibody (WL02495):WB,IHC; JNK antibody (WL01295):WB,IHC; p-JNK (Thr183/Tyr185) antibody (WL01813):WB.

Eukaryotic cell lines

Policy information about cell lines and Sex and Gender in Research

Cell line source(s)

HepG2 and HEK 293T cells were obtained from the iCell Bioscience Inc. (Shanghai China). Primary hepatocytes were derived from different genotypes of 8-week-old male mice.

Authentication

STR authentication

Mycoplasma contamination

Mycoplasma contamination is nagtive

Commonly misidentified lines (See ICLAC register)

No commonly misidentified cell lines were used in the study

Animals and other research organisms

Policy information about studies involving animals; ARRIVE guidelines recommended for reporting animal research, and Sex and Gender in Research

Laboratory animals

We used three genotypes of mice in this study: WT (C57/BL6), LBP -/- (C57/BL6 background), and LBP KI/KI (C57/BL6 background). All mice were initiated in the experiments at 8-week-old. All animals were housed under standard conditions with a temperature of 23 degree centigrade ?, humidity at 60%, and unlimited access to water.

Wild animals

The study did not involve wild animals

Reporting on sex

This study was conducted exclusively using male mice, as female mice are not suitable for investigating lipid metabolism. Estrogen, one of the primary female hormones, exerts considerable influences on fat distribution, lipid metabolism, and insulin sensitivity. By focusing solely on male mice, we aimed to control for confounding factors and establish a clear understanding of the effects of LBP.

Field-collected samples

No field collected samples were used in the study

Ethics oversight	All animal experiments were approved by the Animal Care and Use Committee of the First Affiliated Hospital of China University of Science and Technology.

Note that full information on the approval of the study protocol must also be provided in the manuscript.
